# Engineering bi-directional microstructural heterogeneity through mechanical surface treatment

**DOI:** 10.1016/j.matdes.2026.116654

**Published:** 2026-09

**Authors:** Erencan Oranli, Asghar Heydari Astaraee, Marc Novelli, Thierry Grosdidier, Mario Guagliano, Sara Bagherifard

**Affiliations:** a Politecnico di Milano, Via G. La Masa 1, 20156 Milan, Italy; b Université de Lorraine, CNRS, Arts et Métiers Institute of Technology, Laboratoire d’Etude des Microstructures et de Mécanique des Matériaux (LEM3), Metz F-57000, France

**Keywords:** Ultrasonic shot peening, Heterogeneous materials, Austenitic stainless steel, Localized surface treatment, Severe plastic deformation

## Abstract

By integrating regions with distinct mechanical properties, heterogeneous materials can offer a wide range of mechanical properties and may even surpass the mechanical and functional properties observed in their homogeneous counterparts. Impact-based mechanical surface treatments have proven highly effective in generating microstructural heterogeneity by introducing severe plastic deformation at the surface and subsurface layers, resulting in gradient grain refinement and microstructural modifications while preserving the original properties of the core material. Herein, we employed a strategically modulated ultrasonic shot peening (USP) technique to engineer materials exhibiting bi-directional heterogeneity, i.e., both planar (across the specimen surface) and transversal (in-depth), specifically by creating gradients in both grain size and geometrically necessary dislocation (GND) density. The innovative localized treatment uses masks of various geometric patterns to selectively shield or expose designated regions of the specimens during USP treatment. Tensile tests performed on the USPed specimens evidenced modulated material strengthening, supported by site-specific variations in microstructural features, microhardness, and residual stress distribution. The findings indicate that this novel USP strategy can be implemented to enable engineering materials with heterogeneous structures and tailored mechanical characteristics.

## Highlights

• Selective ultrasonic shot peening (USP) was applied on thin 316L stainless steel samples.
• Bi-directional microstructural heterogeneity was achieved by a selective masking strategy.
• Localized USP created bi-directional grain size/GND gradients that controlled strain localization.

## Graphical abstract

**Figure:**
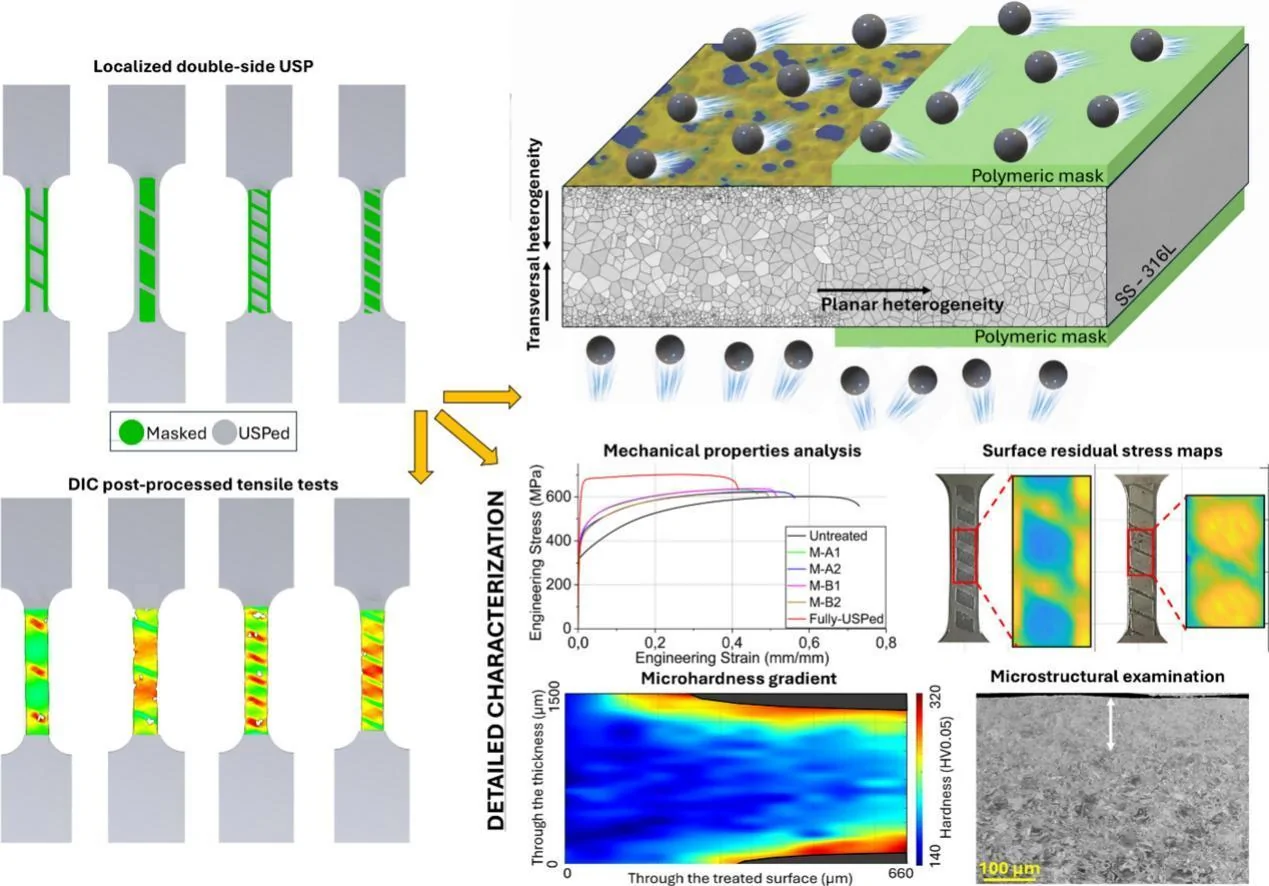

## 1 Introduction

Over recent years, the interest in materials with heterogeneous properties has grown substantially due to the potential customization as well as the superior performance exhibited in some cases compared to homogeneous materials. Unlike high-strength homogeneous materials that tend to produce a limited response under plastic deformation, heterogeneous materials can be modulated to exhibit a wide range of strength and ductility combinations. These materials are characterized by varying mechanical properties in different regions, influenced by the distinguishing mechanical characteristics of the heterogeneous features [1]. Heterogeneity in materials has been implemented in terms of i) phase composition (composite materials) [2], ii) geometrical design (lattice structures) [3], [4], and iii) grain structure (harmonic or bimodal structures) [5].

Although heterogeneous materials have attracted considerable attention and their theoretical behavior is well understood [1], [6], [7], [8], [9], their practical realization remains challenging due to limitations in fabrication scalability and material selection. Studies on materials with microstructural heterogeneity have reported promising results using powder metallurgy approaches [10], [11]. In these studies, mechanical milling was employed to create grain size heterogeneity in spherical powder particles, consisting of coarse grains in the inner region and fine grains near the outer surface. Another manufacturing strategy has been to use asymmetrical rolling on pure Ti to obtain a heterogeneous lamella structures where hard and ultrafine-grained structures embedded the soft and micro-grained structure, leading to extraordinary strengthening [6]. Our group used cold spray deposition as an additive manufacturing method to establish a bimodal (micro-nano) microstructure of a Cu alloy [12]. The results put forward the importance of the powder quality as well as the volume fraction of microcrystalline and nanocrystalline constituents for modulating the overall mechanical performance of the specimens.

Focusing on top-down, also known as transport-based techniques (in contrast to bottom-up constructive processing methods such as powder metallurgy and additive manufacturing), mechanical surface treatments have also been reported to successfully induce microstructural heterogeneity, although mainly limited through depth. These treatments can be used to strengthen materials by inducing a grain-size gradient through severe plastic deformation of the surface and subsurface layers. For instance, Shi et al. [13] produced gradient structures on commercial low-alloying steel through consecutive steps of torsion (twisted between 180°-1440°) and thermal annealing (15 min at 550–600 °C). The resulting microstructure demonstrated a gradient grain size and exhibited hetero-deformation induced (HDI) strengthening. Peening-based surface treatments have also proved effective in this area by forming a nanocrystalline layer at the treated surface, transitioning to larger grains at greater depths depending on the specific peening technique [14]. The resulting gradients can involve several microstructural features, including grain size, twinning volume fraction, constituent phase distribution, geometrically necessary dislocation (GND) density, and phase composition. These characteristics gradually transition from refined surface layers to the bulk material in the core [15], [16], [17], [18]. The specific surface treatment, combined with the initial condition of the material, determines the resulting deformation behavior and facilitates the development of one or more gradient characteristics that can be leveraged to tailor material properties at macroscopic scale.

Shot peening (SP) is one of the most exploited surface modification technologies with already proven capabilities in effectively controlling the microstructure and inducing nanocrystallization [19], [20], [21], [22]. Ultrasonic shot peening (USP), also commonly known as surface mechanical attrition treatment (SMAT), represents an advanced surface modification technique derived from the principles of conventional shot peening. While SP is realized with shots mostly lower than 1 mm in diameter, USP is mostly recognized with larger shots compared to SP, with a diameter up to several mm [23]. Instead of using air pressure like SP, USP relies on ultrasonic vibrations created by a piezoelectric transducer from electrical energy. In USP, the shots are contained in a chamber between the specimen and the vibrating tool, hitting the surface from multiple directions. The impact velocity of the shots is not constant as in SP but distributed over a range, yet the average kinetic energy of the shots and the total coverage of the impingements are high enough to make USP a severe plastic deformation (SPD) process. Thus, generally speaking, under the conditions of comparable Almen intensity and coverage, USP provides a lower surface roughness compared to conventional SP, where the shots are normally directed at a constant angle throughout the entire exposure [24]. In addition to microstructural refinement induced by USP/SMAT, recent efforts have focused on extending the process through the use of hybrid shot media systems to change the surface layers’ chemical composition by impregnating it with a secondary phase relevant for target applications like enhanced wear resistance [25], [26]. Beyond these improvements in the tribological behavior, USP/SMAT has also been extensively investigated for its capability to generate heterogeneous surface structures with enhanced mechanical properties. Previous studies [27], [28], [29], [30] showed that this treatment could generate bimodal heterostructures when the total treatment time was kept below one minute, resulting in incomplete surface coverage. As a result, randomly distributed indentations with different diameters and depths were formed due to the multidirectional impacts occurring at various velocities. Partial coverage with randomized arrangement of the indents created by the impacting shots established the necessary contrast to characterize the overall surface deformation as a heterogeneous structure. The obtained results indicated an enhanced combination of strength and ductility for the treated material. However, extending the exposure time led to an increase in strength but a reduction in ductility, attributable to higher coverage and a consequent decrease in the degree of planar heterogeneity [27].

Herein, we design and implement a practical strategy for inducing bi-directional, that is both planar (across the specimen surface) and transversal (in-depth), heterogeneity in terms of grain size and GND density through a modulated USP technique. The material of choice in this study is 316L stainless steel due to its relatively high strain hardening capacity, low to medium stacking fault energy (SFE) that enables several additional strengthening mechanisms, and wide industrial applications. We design a strategic application of local-USP through targeted surface patterning contrary to the randomized partial coverage implemented in the literature [27], [28], [29], [30]. The results in terms of microstructural and mechanical characterization indicate the high potential of this new approach to efficiently modulate the global mechanical response by inducing localized and site-specific properties.

## 2 Materials and methods

### 2.1 Material & surface treatment

**Fig. 1:**
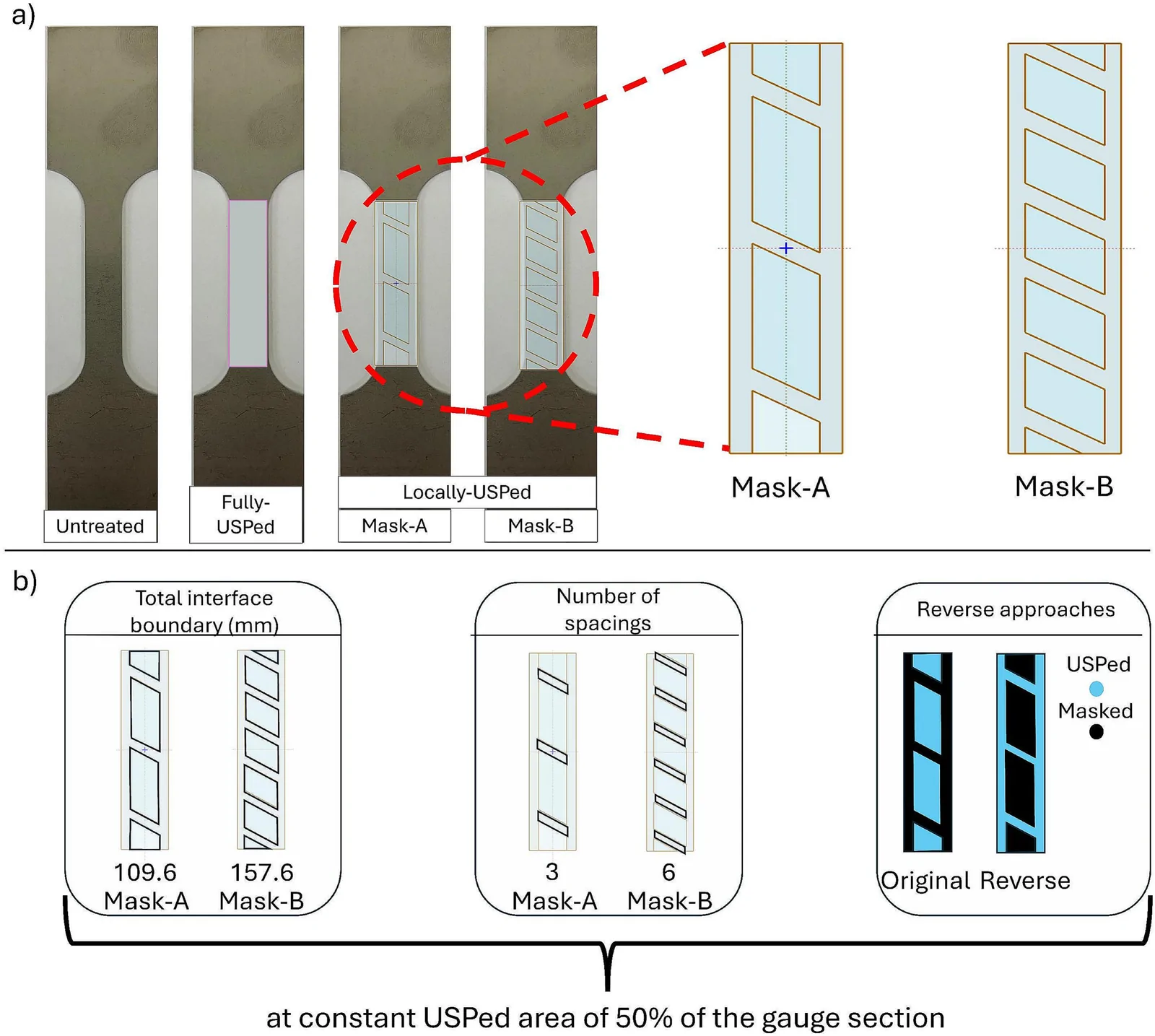
a) Schematic illustrations of the fully- and locally-usped specimens b) different spacings and embedding arrangements on the original and reverse design approaches.

Standard dog-bone tensile test specimens with the gauge section of 42 mm x 10 mm and a thickness of 1.5 mm were laser-cut from an austenitic stainless steel 316L sheet (Migliari Alluminio, IT) and then subjected to USP. Table 1 shows the chemical composition of the material. The USP treatment was performed on both sides of each specimen using Ø2 mm 100Cr6 steel ball bearing shots for a total duration of 10 min on each side using SONATS equipment (Europe Technologies, FR). Ultrasonic frequency was set to 20 kHz, considering a sonotrode amplitude of 60 μm. The selected USP parameters were based on previous investigations on stainless steel, in which similar treatment conditions were shown to effectively induce severe plastic deformation and substantial grain refinement in the surface layers [18], [31], [32]. USP was intermittently applied to both specimen sides in sequential steps of 10 sec (×3 times), 30 sec (×3 times), 2 min (×3 times), and 1 min (×2 times), totaling 10 min of treatment. A 0.7 mm thick industrial tape (Green Belting, CA) was used to mask specific areas for localized USP, with precise cutting performed using a blade cutting machine (Silhouette Portrait, US). To control the extent and arrangement of the areas exposed to USP, two masking strategies—Mask-A (M-A) and Mask-B (M-B)—were implemented. Both strategies employed 28° inclined quadrilateral openings. This inclination was selected as a practical geometrical value to avoid continuous transverse untreated paths across the gauge width, while maintaining overlap of the openings along the gauge length. The openings were arranged in a mirror-symmetric configuration through the specimen thickness. These masks differed in their spacing and the total length of the interface boundaries between peened and unpeened zones, as shown in Fig. 1. Both designs targeted an approximate peening fraction of 50% along the specimen gauge section. However, the effective peened area varied slightly due to differences in pattern spacing and tape placement. The precise effective peening fractions, determined through image analysis, are summarized in Table 2.

**Table 1:** Chemical composition of 316L stainless steel.

Material	C% (Max.)	Si% (Max.)	Mn% (Max.)	P% (Max.)	S% (Max.)	Cr%	N% (Max.)	Ni%	Mo%	Fe%
316L	0.03	1.00	2.00	0.045	0.03	16.5–18.5	0.11	10.0–13.0	2.0–2.5	Balance

**Table 2:** Specimens’ categorization.

Specimen code	Mask type	Total interface boundary length (mm)	Number of spacings through the gauge length	Embedding pairs	Effective peening surface fraction (%)
M-A1	A-original	109.6	3	USPed zones embedded by untreated zones	48.3 ± 1.2
M-A2	A-reverse	109.6	3	Untreated zones embedded by USPed zones	45.6 ± 0.2
M-B1	B-original	157.6	6	USPed zones embedded by untreated zones	42.6 ± 1.8
M-B2	B-reverse	157.6	6	Untreated zones embedded by USPed zones	45.6 ± 0.9

**Fig. 2:**
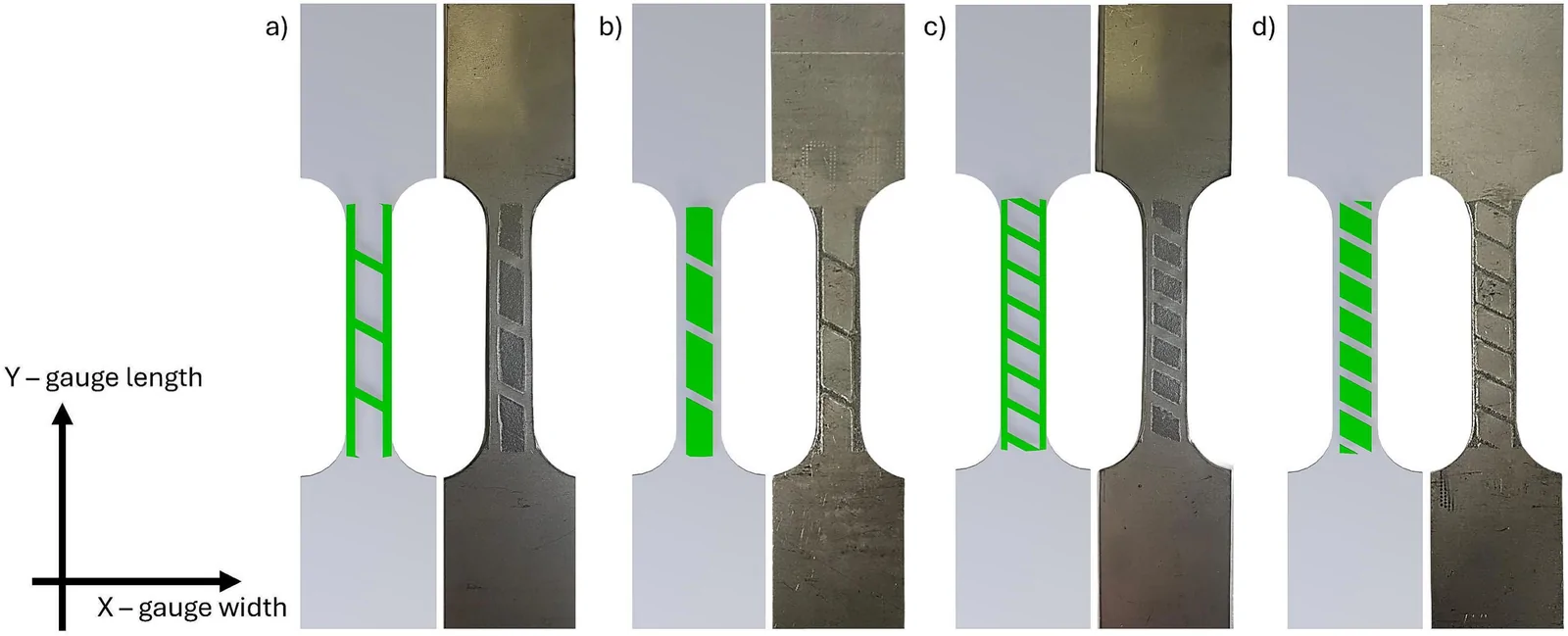
Projected design of the green-coloured masks (left) and their implementation (right) presented on the locally-USPed specimens of a) M-A1, b) M-A2, c) M-B1, and d) M-B2. (For interpretation of the references to colour in this figure legend, the reader is referred to the web version of this article.)

To study the effect of USPed regions’ placement, each masking pattern was also applied in reverse, swapping the masked and unmasked areas. Specimens were labeled “A” or “B” according to the mask type and “1” or “2” to indicate the original or reverse masking strategy. In the original setup, USPed zones were surrounded by untreated regions; in the reverse setup, untreated zones were surrounded by USPed regions, as illustrated in Fig. 1.The implementation of the two masking designs for the original and reverse strategies is presented in Fig. 2.

The Almen intensity measurements were conducted using the standard Almen A strips to evaluate the intensity of the USP treatment. It is a standard index commonly used in peening-based treatments as a measure of the kinetic energy of the process. Almen intensity was set to 16.2A (1/1000 in.). Another key process variable in peening treatments is the surface coverage, defined as the ratio of the plastically deformed area to the total surface area subjected to peening. Short-duration USP trials were conducted under the same processing parameters to estimate the time required for full surface coverage. The treated surfaces were inspected after different exposure times, and full coverage was identified when the surface was almost completely covered by overlapping impact marks. Under the selected conditions, approximately 15 s were required to reach full coverage. Consequently, the 10 min USP treatment corresponded to an effective cumulative coverage of roughly 4000% on each treated face.

### 2.2 Characterization experiments

The protective effect induced by the tapes was preliminarily assessed by projecting residual stress distributions on the untreated and peened surfaces. Surface residual stresses were mapped on a wider area using Pulstec μ-X360J Portable X-ray Residual Stress Analyzer with a Ø1 mm beam inclined to 30°, measured on the {311} austenite plane with a Cr source to check the planar distribution.

Microstructural characterization was done by Jeol F100 scanning electron microscope (SEM) while ATEX software (Version 4.14, FR) [33] was used to post-process the data. For microstructural characterization, a segment from the USPed area of each series was sectioned and mounted for better manipulation. The specimen surface preparation was launched with coarse grinding to P1200 grit, then proceeded with two stages of fine mechanical polishing using 9 µm and 3 µm diamond suspensions. The final step involved mechanical and chemical polishing with a 250 nm silica suspension. Electron backscatter diffraction (EBSD) analysis was performed on the cross-section with step sizes of 250 and 100 nm. Gradient functions were plotted in terms of GND density to further investigate the deformed and refined layers of the microstructure. Deformation-induced twins were detected and mapped as coincidence site lattice structures, identified as Σ3 boundaries with a misorientation angle of ∼60° on the 〈111〉 plane.

Microhardness tests were performed with a Zwick Roell Vickers hardness tester on the specimen’s cross-sections at a load of 50 gf and a dwell time of 15 s. The tests were repeated three times to assess the repeatability of the hardness profile.

Uniaxial tensile tests were conducted at a crosshead speed of 2 mm/min using an MTS Alliance RT/100 testing machine. The deformation and strain localization during tensile tests were analyzed using ARAMIS High-Speed Digital Image Correlation (DIC) Systems (Zeiss, DE). Image acquisition was conducted at a frequency of 1 Hz, capturing the specimen’s surface. GOM Correlate 2020 software (2020 Hotfix 4) was used to post-process the DIC images. The test was repeated three times for each configuration, including the untreated, fully-USPed, and locally-USPed series.

## 3 Results and discussion

### 3.1 Verification of the tape protection

**Fig. 3:**
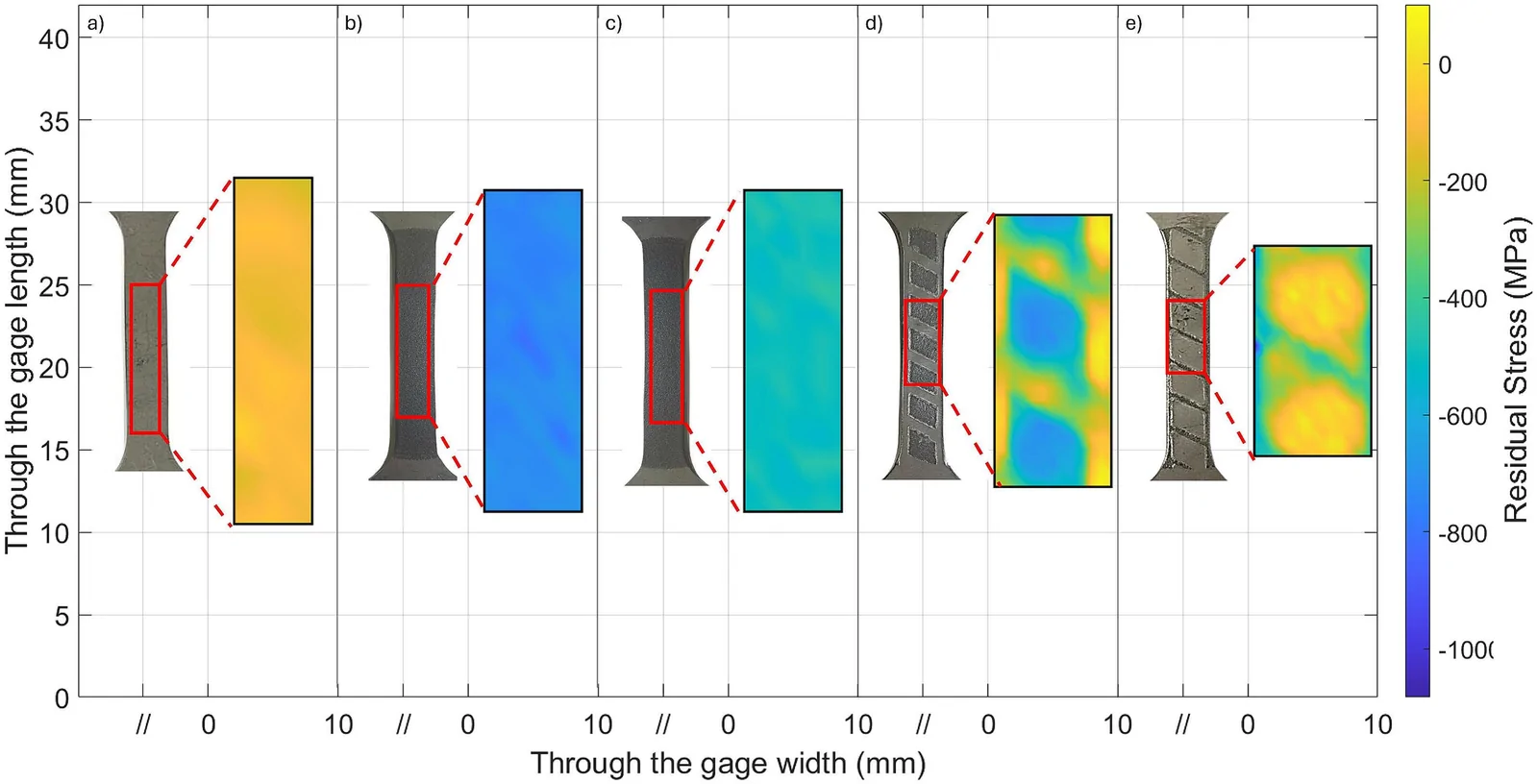
Longitudinal in-plane surface residual stress maps for; a) untreated specimen, b) front face of fully-USPed specimen, c) back face of fully-USPed specimen, d) front face of M-B1 specimen, and e) back face of M-B2 specimen.

The surface residual stress profiles have been characterized in terms of the longitudinal in-plane stress component (parallel to Y-direction in Fig. 2). The specimen faces were denoted as front and back, with the front face corresponding to the side peened first during the USP sequence. Fig. 3a–e shows the surface heatmaps of the same stress component for the untreated, fully-USPed (front face), fully-USPed (back face), M-B1 (front face), and M-B2 (back face) specimens. The untreated condition was mapped over an area of 21 mm × 6 mm using a 3 mm step size between adjacent measurement points. The front and back faces of the fully-USPed specimen were mapped over an area of 19.5 mm × 7.5 mm using a 1.5 mm step size. For the locally-USPed conditions, a finer step size of 0.75 mm was employed to better resolve the local residual stress variation between USPed and masked regions. The mapped areas were 16.5 mm × 9 mm for the front face of the M-B1 and 12.75 mm × 9 mm for the back face of M-B2. In Fig. 3a, the untreated specimen’s surface stress map reveals a consistent compressive pre-stress of about 100 MPa, likely resulting from the sheet manufacturing process. Although laser cutting may introduce localized thermal residual stresses near the cut edges, all specimens were prepared using the same cutting procedure. Moreover, the untreated gauge region showed a nearly homogeneous compressive pre-stress of approximately 100 MPa, which was substantially lower than the compressive residual stresses induced by USP. Therefore, the initial cutting-related stress state was considered a common baseline condition and was not expected to govern the comparative response of the USPed specimens. The results indicate a clear difference in the residual stress patterns between the front and back faces of the fully-USPed specimen, with average values of −700 MPa and −450 MPa, respectively (Fig. 3b and c). These variations could arise from changes in material properties linked to the intermittent sequential USP steps on the opposite side. With each USP pass, the opposite side of the material also undergoes some hardening, leading to varied deformation response over the sequential USP steps and thus uneven residual stresses across the specimen faces. For the locally-USPed M-B1 and M-B2 specimens, the residual stress maps reflect the respective mask designs: the USPed regions had stress values similar to those in the same corresponding face of the fully-USPed specimen (Fig. 3b–c), while the untreated regions had stress levels close to those of the untreated specimen (Fig. 3a). Therefore, the front/back residual stress asymmetry introduced by sequential USP was consistently preserved in both the fully- and locally-USPed specimens and is considered to have a limited influence on their comparative mechanical response. The surface residual stress analysis also demonstrates that the protective tape provided an effective local protection against the effect of the peening shots.

### 3.2 Microstructural analysis

**Fig. 4:**
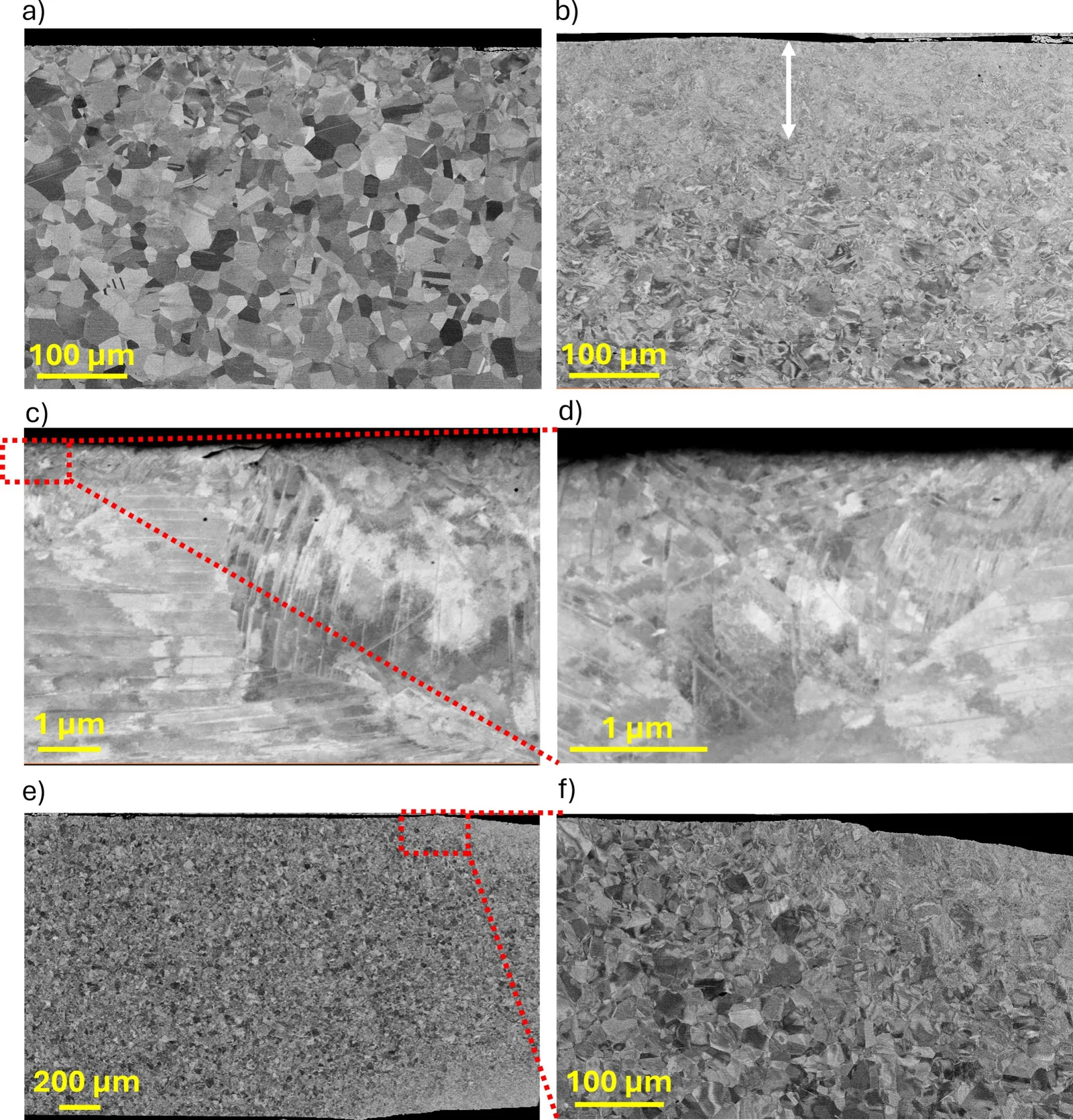
Cross-sectional micrographs of a) the untreated specimen, b,c,d) the fully-USPed specimen at different magnifications, e,f) the locally-USPed specimen (M-A1) at different magnifications.

SEM analysis on the specimen cross-sections revealed the significant effect of plastic deformation on the microstructure. As shown in Fig. 4a, the microstructure before USP is characterized by equiaxed grains with a relatively homogeneous grain size distribution with an average grain size of 18 ± 2 μm. Fig. 4b exhibits the microstructure of the fully-USPed specimen, which consisted of a continuous affected top layer with significantly deformed/refined grains in the topmost surface, while deeper regions show less-refined, yet still affected grains. The thickness of the grain refined layer was estimated to be 100–150 μm from SEM images based on contrast homogeneity: the refined surface layer exhibits relatively uniform contrast as highlighted by the white arrow in the corresponding figure, whereas the underlying region shows greater contrast variation associated with coarser grains.

Fig. 4c shows the microstructure at the extreme surface of the USPed zone. Microstructural planar defects with an average width of ∼200 nm were observed on the left side of Fig. 4c whereas on the right side of Fig. 4c, refined blocks are observed. Based on previous studies, these features are likely associated with nanoscale shear bands [34] and/or twin-twin interactions [35]. Grain refinement phenomena can be initialized through dislocation slip or mechanical twinning [36]. The level of SFE of the material leads to one of the two mechanisms being dominant. As a low to medium SFE material, 316L austenitic stainless steel has the potential of twinning as the main mechanism. Finally, a thin and consistent layer made up of randomly oriented sub-micron grains (less than 200 nm) was developed on the specimen’s surface, as shown in Fig. 4d. This observation aligns with previous studies [18], [34], [35], [37], [38], which describe the grain refinement process by peening-based treatments in 316L stainless steel occurring through the following main stages: i) the formation of mechanical micro-twins or nanoscale shear bands; ii) the creation of refined blocks due to intersections between twins; and iii) the development of randomly oriented nano-grains as a result of grain boundary sliding or rotation.

Fig. 4e presents the cross-sectional microstructure of the locally USPed M-A1 specimen. Pronounced grain refinement is observed near both treated surfaces, with a gradual transition toward the coarser core microstructure. This confirms the formation of microstructural heterogeneity, both through the thickness and along the surface plane. A higher magnification view of the interface between the USPed and masked regions is provided in Fig. 4f. Beneath the protected region, the surface grains largely retain their original dimensions, whereas the adjacent USPed region shows pronounced refinement. The relatively sharp yet continuous planar transition between the peened and masked regions demonstrates the capability of localized USP to generate controlled bi-directional heterogeneity.

**Fig. 5:**
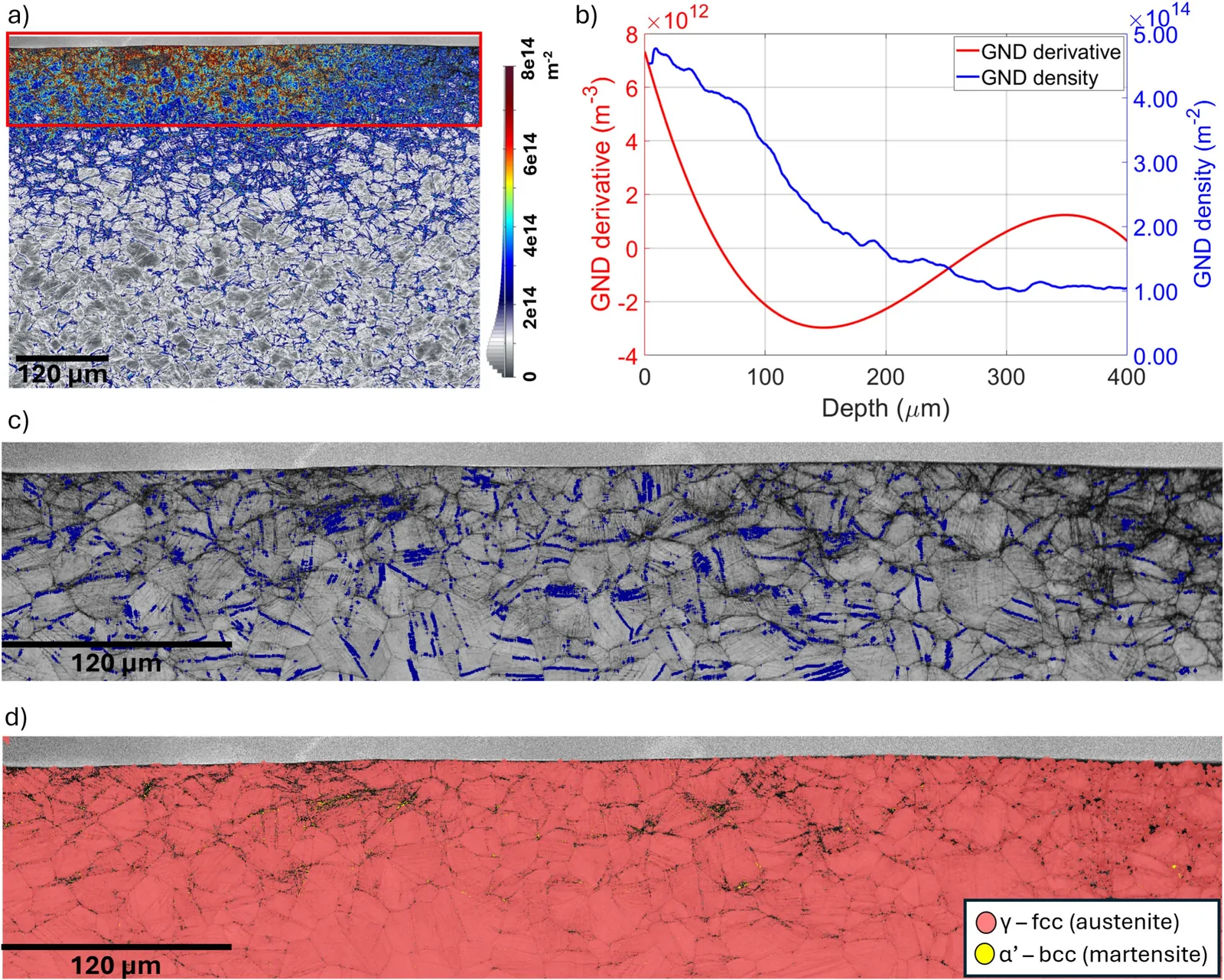
A) ebsd-based gnd density map of the fully-usped specimen cross-section and b) corresponding in-depth gnd profile through the depth of fully-usped specimen c) coincidence site lattice structures (σ3 boundaries ∼ 60° – 〈111〉 plane) detected by EBSD close to the surface layer projected on the band contrast map, d) EBSD phase map, where red pixels indicate γ-fcc austenite and yellow pixels indicate α’-bcc martensite. (For interpretation of the references to colour in this figure legend, the reader is referred to the web version of this article.)

The results of the EBSD analysis performed on the cross-section of the fully-USPed specimen are shown in Fig. 5. The GND density distribution over the USPed cross-section is depicted in Fig. 5a-b, while Fig. 5c shows the subsurface cross-section extracted from the band contrast image with projected Σ3 twin boundaries (60° misorientation and about 〈111〉 plane).

A significant accumulation of GNDs is evident at the surface and sub-surface regions, as their density decreased progressively with depth (Fig. 5a). Several dark, non-indexed regions were observed close to the surface. These regions are attributed to sub-step-size features (∼250 nm) and SPD, which reduced diffraction quality and prevented reliable indexing due to pattern superposition. The overall indexation rate was 96.4%, but the first 3 µm from the surface showed poor indexation, as reflected by the data plots where consistent measurements began only beyond this depth. The deformation state on the cross-sections showed some heterogeneity in the spatial distribution of GNDs in the direction parallel to the surface. The average GND density plotted as a function of depth in Fig. 5b, indicates a decreasing trend for GND from the surface to the core material, which is caused by a similar decrease in the effective strain. The GND-rich layer extends to a depth of approximately 300 µm, where it plateaus with no significant changes.

The derivative of the GND density based on a polynomial function is also plotted on the same graph; this parameter is normally used to visualize the extent of grain-refined regions through the thickness of surface treated materials [39]. The initial nullification point of the derivative, which corresponds to a depth of 70 µm in this case, is normally considered the maximum threshold at which refined microstructures, characterized by sub-grain structures, are formed. Whereas the second nullification point, corresponding to 290 µm, refers to the thickness of the total affected layer [39].

Fig. 5c shows the EBSD twin boundary map projected over the band contrast map of the fully-USPed substrate, corresponding to the region highlighted by the red bounding box in Fig. 5a. A high density of coincidence site lattice Σ3 boundaries characterized by a misorientation angle of ∼60° about 〈111〉 plane (highlighted in blue) is observed within the near-surface layer, indicating the presence of deformation-induced mechanical twins. Their pronounced fraction in this region, together with the elevated GND density gradient, suggests that SPD promoted twinning as the primary grain-refinement mechanism. This observation supports the interpretation of the microstructural planar defects in Fig. 4c as twin-related structures.

Because deformation-induced martensite formation has been reported in USP-treated stainless steel [37], [40], EBSD phase analysis was also performed, as indicated in Fig. 5d. The phase map indicates that martensitic transformation was negligible under the present treatment conditions, with an average α′-bcc martensite fraction of approximately 0.25% in the first 75 µm beneath the surface. The few martensite pixels were mainly located near grain-boundary-rich regions. Therefore, the strengthening contribution from martensitic transformation was considered negligible compared with the effects of grain refinement, GND accumulation, deformation twinning, and work hardening, consistent with previous observations for similar conditions [18], [34].

**Fig. 6:**
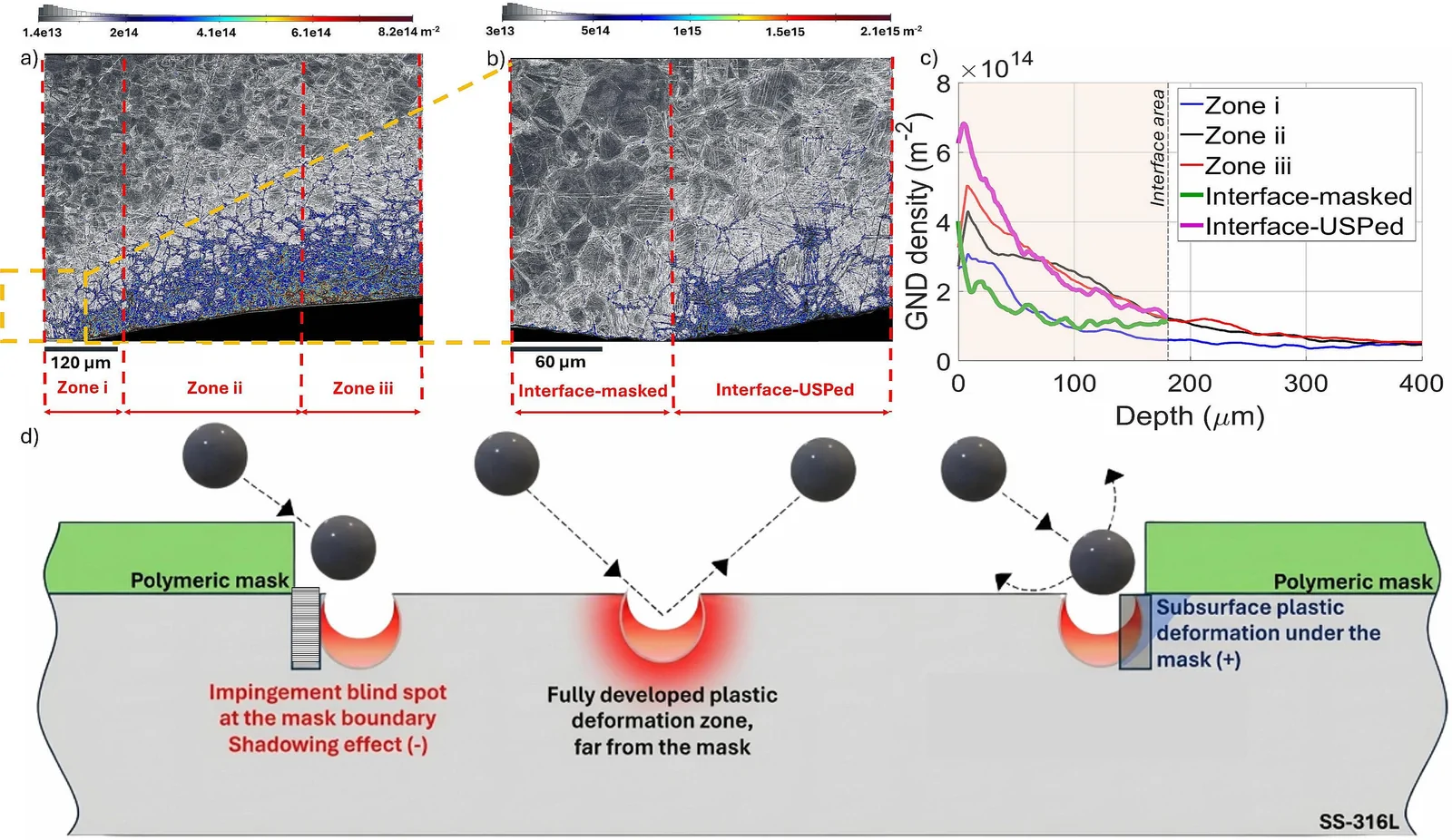
GND density variation across the M-A1 locally-USPed specimen’s cross section a,b) EBSD-based GND density maps; and c) corresponding in-depth GND density profiles; d) schematic representation of the shadowing effect and the extension of plastic deformation under the mask.

EBSD analysis was also conducted on the M-A1 locally-USPed specimen at the interface between the USPed and the masked zones. The results are shown in Fig. 6 in terms of GND density (Fig. 6a–c), while Fig. 6d shows the visual representations of the deformation zones close to and far from the mask boundary. It should be noted that the tape protection prevented direct shot impacts on the masked regions, as confirmed by residual stress mapping (Fig. 3); however, localized deformation could still develop near the mask boundary due to stress transfer from adjacent USPed regions. Therefore, the USPed/masked interface should be interpreted as a transition zone rather than an abrupt boundary between fully deformed and completely unaffected material. For quantitative analysis the micrograph at 200× magnification with a scanning step size of 250 nm (Fig. 6a) was partitioned into three sections, i) the edge region located at the interface between the mask and USPed area, ii) the transition region that represents a zone of gradual microstructural transition and is characterized by a slight lateral contraction of the outer surface as a result of the USP treatment and iii) the USPed region, which is sufficiently distant from the masked surface [39].

A higher magnification analysis at 500× was focused on the masked zone and the adjacent USPed zone, constructing the two halves of the exact interface between the masked and peened regions, using a smaller step size of 100 nm (Fig. 6b). GND accumulations were investigated in these areas to analyze the microstructural feature’s gradient along the depth, as plotted in Fig. 6c. GND pile-ups were generally abundant within zone iii, whereas zone i exhibited a noticeable reduction in GND density. In contrast, the masked region at the interface, especially at subsurface levels, demonstrated a notable decrease in GND concentrations, as plotted in Fig. 6b. The accumulation of GNDs beneath the masked region revealed that local plastic deformation occurred due to stress transfer from adjacent impacted zones, leading to slight microstructural changes even where direct shot impacts were absent as illustrated in Fig. 6d.

The GND density observed in zone iii of the locally-USPed specimen corresponds closely to the GND density detected in the fully-USPed specimen. Among the analyzed regions in the lower-magnification assessment, zone iii exhibited the greatest GND density, followed by zone ii, with zone i displaying the lowest values. Notably, the USPed half of the interface demonstrated the highest GND accumulation, particularly near the free surface. This result is primarily attributed to the enhanced resolution of EBSD analysis achieved through a finer scanning step size, which enabled the identification of dislocation features with dimensions ranging from 100 nm to 250 nm. The variations in GND density show the relatively non-homogeneous behavior of plastic deformation in the region close to the mask and how the presence of the mask influenced the deformation state and, thus, grain refinement. The results confirm that the presence of a mask with a certain thickness (0.7 mm) can induce a shadow effect and locally limit the number of impingements on the edges. For graphical representation to facilitate a clearer understanding of the shadowing effect, the region nearest to the mask boundary—interpreted as the impingement blind spot—is highlighted by a dashed rectangular box in Fig. 6d.

GND density showed a strong peak around the depth of 15 μm in all the investigated zones except for the masked zone, as expected. This peak is followed by a decreasing trend towards the surface in the first 15 μm of the material and can be attributed to two main phenomena: i) a portion of GND populations is still not identified, particularly immediately below the free surface where ultrafine grains are formed but the step size is limited to 100 nm, ii) GND accumulations start transforming into low-angle grain boundaries (LAGB) at increased strains due to the ongoing grain refinement. In the latter scenario, further increase in misorientation can cause transformation of LAGB into high-angle grain boundaries (HAGB) and eventually result in a decrease in GND density [41], [42]. Instead, the plastic deformation extent in the masked zone was not sufficient to induce such transformations and thus the GND density kept increasing towards the free surface.

### 3.3 Microhardness measurements

**Fig. 7:**
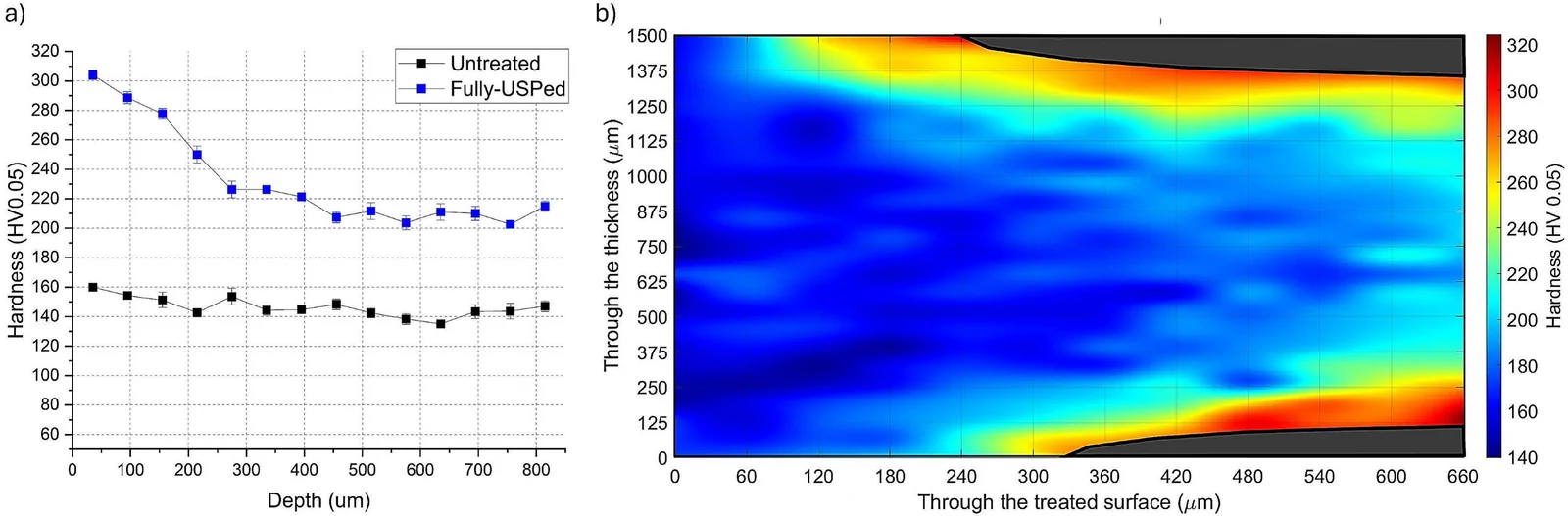
In-depth hardness profiles of untreated and fully-USPed 316L stainless steel: a) hardness profiles from the surface to the core, b) hardness heatmap across the transition region between USPed and masked zones in the M-A1 locally-USPed specimen.

Microhardness was measured from the surface to 800 μm to assess the hardened layer depth. Fig. 7a shows the hardness profile for the untreated and fully-USPed specimens. The hardness immediately below the USPed surface was measured to be 305 HV, recording a 90% increase compared to the surface hardness of the untreated specimen (160 HV). The stabilized hardness in the core of the fully-USPed specimen was around 210 HV, significantly higher than the core hardness of the untreated state, measured to be 140–150 HV. This observation confirmed that the work hardening occurred throughout the entire specimen thickness. The hardness gradient of the fully-USPed specimen stabilized at a depth of ∼300 μm, coinciding with the subsurface layer characterized by a high density of GND features.

Further microhardness mapping was performed on a window of 660 × 1380 μm^2^, on a cross-section at the interface of USPed and masked zones of the M-A1 locally-USPed specimen, as shown in Fig. 7b. The heatmap was generated from 12 linear Vickers microhardness profiles measured across the interface region using the same load and dwell time reported in Section 2.2. The measurement points were arranged to cover comparable material volumes on both sides of the tape-protected boundary. To avoid interaction between adjacent indentation plastic zones, the spacing between neighboring indentations was kept at least 2.5 times the indentation diagonal. The hardness map was constructed by interpolation between neighboring valid measurement points. The wider part of the cross-section corresponds to the masked region (left side of Fig. 7b), while the curved and narrower part represents the USPed portion of the cross-section (right side of Fig. 7b). Some asymmetry was observed between the hardness levels of the two opposite USPed faces. This difference could be mainly due to the heterogeneous nature of USP as well as the intrinsic multi-directional and random impact sequences. Additionally, a plausible mechanism contributing to the observed strengthening effect is the sequential application of alternating treatments on the opposite sides of the specimen. This approach likely induced a progressive strengthening and resistance to plastic deformation, whereby each successive treatment incrementally increased the material’s strength to some extent through the thickness. Such repeated peening steps may facilitate cumulative hardening, ultimately resulting in a more robust material through the cross-section.

Similar to what was observed in the fully-USPed specimen (Fig. 7a), the locally-USPed specimens also showed hardening throughout the entire cross-sections. The hardness heatmap revealed that the hardness levels at the specimen’s core area consistently remained higher than those found in the core of the untreated specimen. The microhardness analysis indicated some degree of hardening also under the masked regions, which is grown in the orthogonal direction with respect to the surface treatment direction. This causes the actual total USPed volume to increase with respect to the initial design and could be considered an additional strengthening factor.

### 3.4 Tensile tests

**Fig. 8:**
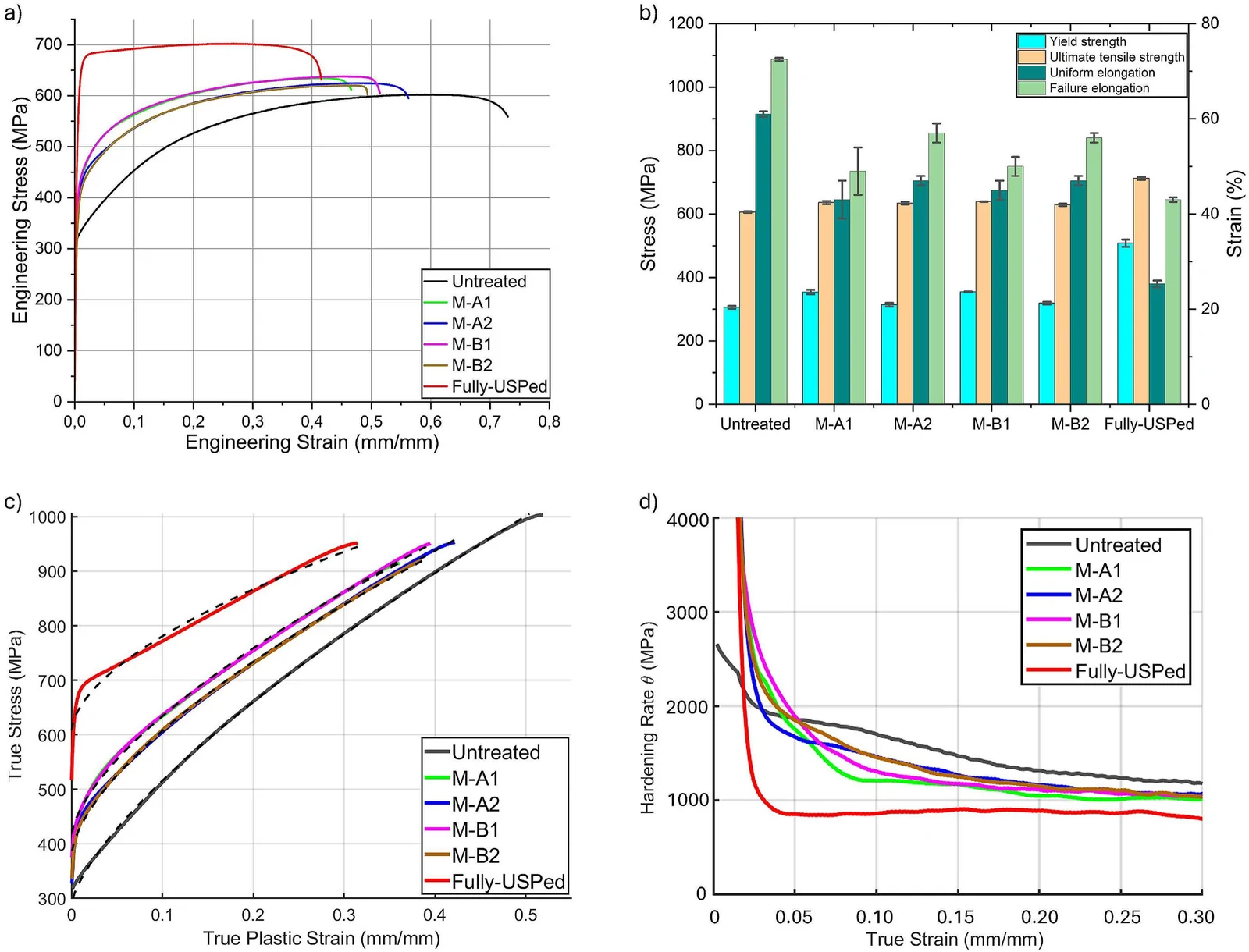
a) Engineering stress–strain curves for the untreated and all the USPed conditions, b) comparison between major mechanical indexes extracted from the tensile tests, c) true stress vs. true plastic strain curves for all the conditions along with the fitted Ludwik hardening curves (black dashed lines), and d) hardening rate (θ=dσ/dε) curves for the tested series.

The stress–strain curves for all the USPed series as well as the untreated specimen are presented in Fig. 8a. The corresponding mechanical properties, including 0.2% offset yield strength (YS), ultimate tensile strength (UTS), uniform elongation, and elongation to failure, are summarized in Fig. 8b and Table 3. Fig. 8a shows a representative stress–strain curve among the three tests performed per series, considering the low deviation observed (Fig. 8b and Table 3). The untreated specimen exhibited the lowest strength and the highest ductility among all the tested series. The fully-USPed specimen, on the other hand, showed the highest relative strengthening by exhibiting a notable improvement in terms of YS and UTS, yet with a 59% reduction in uniform elongation. Such a significant change compared to the untreated specimen promised a large window for modulating the mechanical response in the locally-USPed configurations through geometrical pattern designs. The stress–strain response of the locally-USPed conditions varied in the range between the two extreme conditions of untreated and fully-USPed. All the locally-USPed series exhibited strengthening with loss of ductility compared to the untreated condition. M-A1 and M-B1 specimens showed relatively identical behavior until the onset of necking (i.e., uniform elongation). Similarly, stress–strain curves of M-A2 and M-B2 overlapped until uniform elongation. The original designs (M-A1 and M-B1) resulted in higher strengthening compared to the reverse designs (M-A2 and M-B2).

**Table 3:** Tensile test data for the untreated and USPed specimens.

	Untreated	M-A1	M-A2	M-B1	M-B2	Fully-USPed
YS (MPa)	306 ± 5	354 ± 7	314 ± 6	355 ± 1	319 ± 4	508 ± 12
UTS (MPa)	606 ± 2	636 ± 5	634 ± 4	639 ± 1	629 ± 4	712 ± 5
Uniform elongation (%)	61 ± 0.6	43 ± 4	47 ± 1	45 ± 2	47 ± 1	25 ± 0.7
Elongation to failure (%)	73 ± 0.3	49 ± 5	57 ± 2	50 ± 2	56 ± 1	43 ± 0.5

The total interface boundary between USPed and masked domains was planned to be different for the A and B designs. As described previously, the M-B local-USP design was characterized by 43% higher total boundary length compared to the M-A design. This has to be noted because some deformation expanded under the mask, thus causing an increase in effective treated fraction, but at the same time, the mask caused some shadowing effect and thus a reduction in effective deformation in the peened zone (Fig. 6b and d). Also, such interface boundaries in heterogeneous structures can act as active sites for the creation of extra GNDs by the formation of back-stress [43], [44]. Thus, for interpreting the behavior of locally-USPed specimens, we consider not only the masking geometry, but also the effective peening fraction (that slightly varies between specimens as indicated in Table 2) and more importantly the total USPed / unUSPed interface boundary.

In general, the locally-USPed specimens exhibited strength-elongation values between those of the untreated and fully-USPed conditions, showing a conventional strength-ductility trade-off. A similar trend was observed among the locally-USPed specimens: the original design approach produced an increase in strength relative to the reverse approach, while exhibiting reduced ductility, as measured by uniform elongation. The total USPed / unUSPed interface boundary, as considered in this study, did not exert a discernible influence on the final response, as the original approach produced nearly identical stress–strain curves for the type A and type B designs up to the onset of uniform elongation. A comparable trend is evident for the reverse approach as well (Fig. 8a and c). The increase in GND population associated with the larger interface boundary was counterbalanced by the reduced peening fraction and the shadowing effect caused by the mask geometry. Although the total interface boundary area in type B was 43% greater than that of type A, this enhancement was insufficient for GND accumulation to outweigh the shadowing effect within the present design.

Fig. 8c shows the true stress-true plastic strain data extracted from tensile tests. The data were fitted using the Ludwik hardening model (Eq. (1)) [45], shown as black dashed lines. The Ludwik model consists of three constant parameters: $\sigma_{0}$ indicates the first yield stress, K the strength factor, and n the strain hardening exponent. The fitted model parameters are summarised in Table 4. All specimens demonstrated excellent regression fits, with R^2^ values exceeding 0.99 for untreated and locally-USPed conditions, and a similarly strong fit for the fully-USPed specimen (R^2^ = 0.97). Significant plastic deformation and the resulting grain refinement created an in-depth strain gradient between the USPed surface and the interior material, even at low overall strains, as seen in the difference between untreated and fully-USPed specimens (Fig. 8c). Fully-USPed specimens created a significant increase (124%) in parameter $\sigma_{0}$, which is the starting point of the fitted Ludwik curve and an indicator of YS. In contrast, the locally-USPed specimens showed only slight variations among themselves, reflecting similar changes to 0.2% offset YS results provided in Table 3.

**Table 4:** Ludwik hardening parameters for the untreated and the USPed conditions.

	$\sigma_{0}$(MPa)	K (MPa)	n	R^2^
Untreated	277 ± 4	1173 ± 17	0.69 ± 0.00	0.99
MA-1	415 ± 6	936 ± 18	0.62 ± 0.03	0.99
MA-2	400 ± 3	1026 ± 7	0.68 ± 0.00	0.99
MB-1	408 ± 3	966 ± 8	0.63 ± 0.01	0.99
MB-2	387 ± 4	1009 ± 8	0.64 ± 0.00	0.99
Fully-USPed	622 ± 8	655 ± 4	0.57 ± 0.01	0.97

The strength coefficient, K, serves as a key measure of a material's ability to harden. Logically, the untreated specimen demonstrates the greatest hardening potential. The plastic deformation introduced by USP leads to GND accumulations at the grain boundaries, which in turn limits the number of dislocations that can contribute to further hardening under tension; this “pre-hardening” effect was most pronounced in the fully-USPed specimens, as evidenced by their lowest K values. Locally-USPed series showed similar K parameters, but the reverse designs (M-A2 and M-B2) exhibited higher K values, indicating better strain hardening when softer regions were englobed by the harder ones. This improvement is also seen in the hardening rate coefficient, n, suggesting that the reverse treatment helped preserve the material’s hardening ability.

(1) $$\sigma =\sigma_{0}\;+K*\varepsilon^{n}$$

To observe how the specimens' ability to harden changed under tensile loading, strain hardening rate (θ = *d*σ/*d*ε) was analyzed as a function of strain. This data is presented in Fig. 8d after applying a Savitzky-Golay filter (polynomial degree of 3). At small strains (ε < 0.05), as plastic deformation started, the fully-USPed specimen exhibited a lower hardening rate than the untreated one. Notably, all locally-USPed specimens exhibited a higher hardening rate than both the untreated and fully-USPed series within this specific strain range. This enhanced hardening is likely due to the buildup of GNDs at the boundaries between the hard and soft domains, which increases dislocation storage. However, at larger strains, the locally-USPed specimens shifted their behavior to fall between that of the untreated and fully-USPed series. A slow-down in the reduction of the hardening rate was recorded for the reverse approach (M-A2 and M-B2) in the early strain levels; this can be attributed to the preserved hardening ability and the increased uniform elongation of these specimens.

**Fig. 9:**
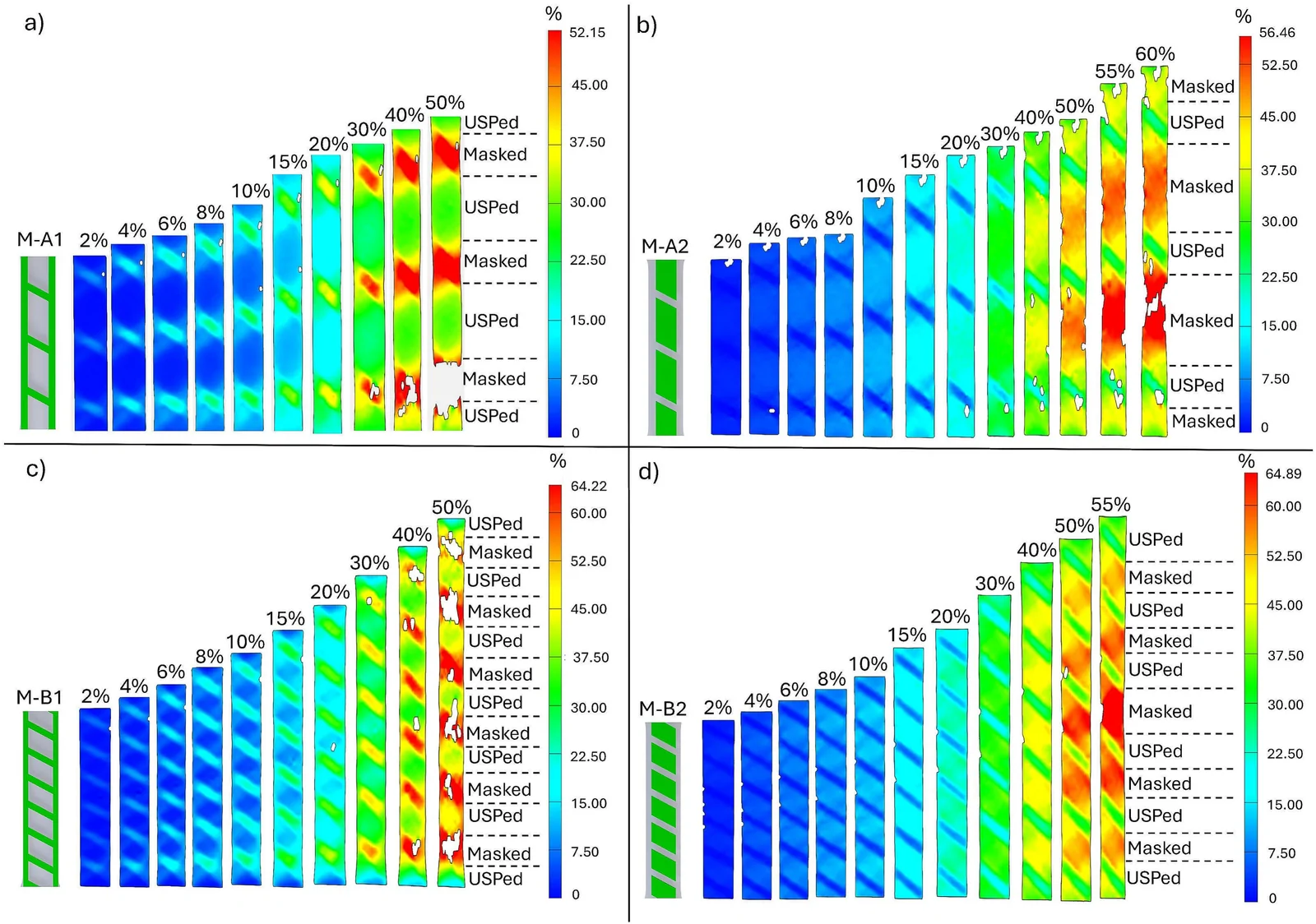
Local strain maps captured by DIC camera at increasing global strain levels for a) M-A1, b) M-A2, c) M-B1 and d) M-B2.

Besides the specimens’ global behavior, the local variation of strains during tensile testing was also analyzed using DIC post-processing. Fig. 9 shows the strain contours of the locally-USPed specimens at certain global strain levels in the overall range of 2–60%. The number above each strain contour indicates the corresponding global strain. To better visualize the contours, only the main gauge sections are shown in Fig. 9.

In the initial phase of deformation (about 2% global strain), all specimens showed early strain localization, mainly in the masked regions. As the strain increased, these localized zones expanded into adjacent areas beyond the original spacing. This effect is especially clear in specimens M-A1 and M-B1, where the localized strain forms a noticeable “X” pattern (see Fig. 9a and 9c at the strain levels starting from 4%). This pattern resulted from the mirror-symmetric mask design on opposite specimen faces, which caused intersecting strain localization across the spacing zones. This led to increased strain localization in the later-forming regions in the original approach, which were influenced by the placement of USPed areas on the opposite side of the specimen. Since both M-A2 and M-B2 used mirror-symmetric mask designs, a similar pattern of intersecting strain contours appeared in these specimens. However, as the peened zone were continous in the reverse approach, USP reduced the strain localization on the opposite side, causing strain delocalization (see Fig. 9b and d). It is to be noted that the DIC system did not provide sufficient spatial resolution to reliably capture strain fields at the extreme edges. Consequently, the outermost portions of the specimens were excluded from Fig. 9. Due to the limited data quality in these areas, strain contours could not be resolved, and no clear indication of strain localization was detected at the edges.

Analysis of the strain contours reveals that reverse design approach (M-A2 and M-B2), characterized by a continuous hardened USPed exterior enclosing softer untreated regions, showed a more uniform strain distribution during early global strain stages compared to the corresponding original configurations (M-A1 and M-B1). At a global strain of 30%, specimens with reverse design exhibited reduced strain localization. Embedding untreated, more ductile zones within a hardened matrix was found to lead to improved ductility. These findings are consistent with earlier studies, which reported increased uniform elongation in heterogeneous structures where softer zones are englobed within the harder ones [6].

**Fig. 10:**
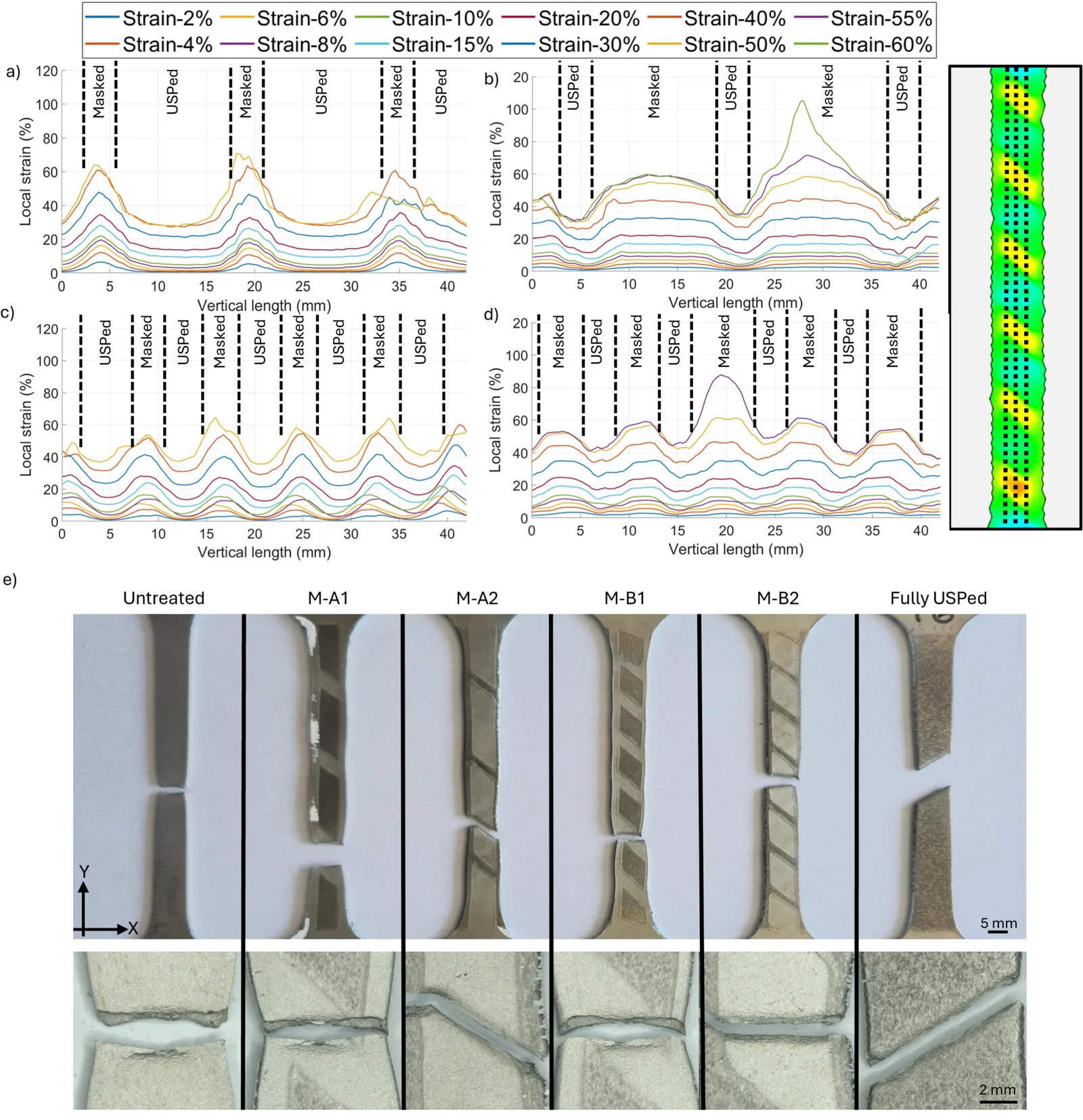
Vertical local strain profiles averaged over five linear profiles for each specimen of a) M-A1, b) M-A2, c) M-B1 and d) M-B2 designs, e) macroscopic examination of the fractured tensile specimens; full gauge section (upper row), and magnified fracture sections (lower row).

Focusing specifically on how strain varies between masked and unmasked regions, Fig. 10 presents the local strain profiles for the locally-USPed specimens, averaged based on three linear paths along the specimen’s length (as shown by the dashed lines in the representative schematic on the right side of Fig. 10). Reliable strain data was obtained up to 50% global strain for M-A1 and M-B1, and up to 55% and 60% for M-B2 and M-A2, respectively. In the M-A2 specimen, more pronounced strain difference between USPed and masked areas appeared only after the global strain exceeded 10%, which was later than in the other locally-USPed specimens. This confirms again the favourable effect of confining the softer untreated area within the USPed zones for maintaining plastic deformation, as the larger area available for local strain buildup allowed stress to spread out, resulting in a more even strain distribution. In contrast, for the M-A1 specimen, these strain differences appeared much earlier.

M-A2 and M-B2 specimens (the reverse approach) showed less pronounced strain peaks during later stages of deformation (from the end of uniform elongation to final failure), indicating more stable failure behavior (Fig. 10b, d), compared to the original designs (M-A1 and M-B1) (Fig. 10a, c). Additionally, the more pronounced relative difference in elongation at failure between the reverse and original approaches, as compared to their respective uniform elongation differences (see Table 3), further confirms this observation. A macroscopic examination of the fractured tensile specimens was carried out, as shown in Fig. 10e. The upper row shows the full gauge sections after fracture, while the lower row provides the isolated and magnified views of the fracture regions. Since the specimens were tested along the longitudinal direction, the masked spacings in M-A1 and M-B1 extended along the Y-direction, forming horizontal paths passing fully across soft zones across the lateral cross-sections at higher strain levels (see Fig. 9a and c). The final fracture locations in these specimens were consistent with these highly strained regions, suggesting preferential failure through the connected softer zones. The fully homogeneous soft sections increased strain localization at increased global strain, which had previously been restrained by the pattern geometry. On the other hand, the reverse designs (M-A2 and M-B2) did not lead to the formation of such homogeneous cross-sections, resulting in enhanced resistance up to final fracture. Their fracture surfaces exhibited inclined failure paths near the specimen edges, similar to the fully-USPed condition, indicating a comparable crack propagation behavior in the edge regions subjected to USP. Overall, the fracture observations are in good agreement with the DIC strain maps, confirming that the final failure locations corresponded to the regions experiencing the highest local strain during tensile loading.

**Fig. 11:**
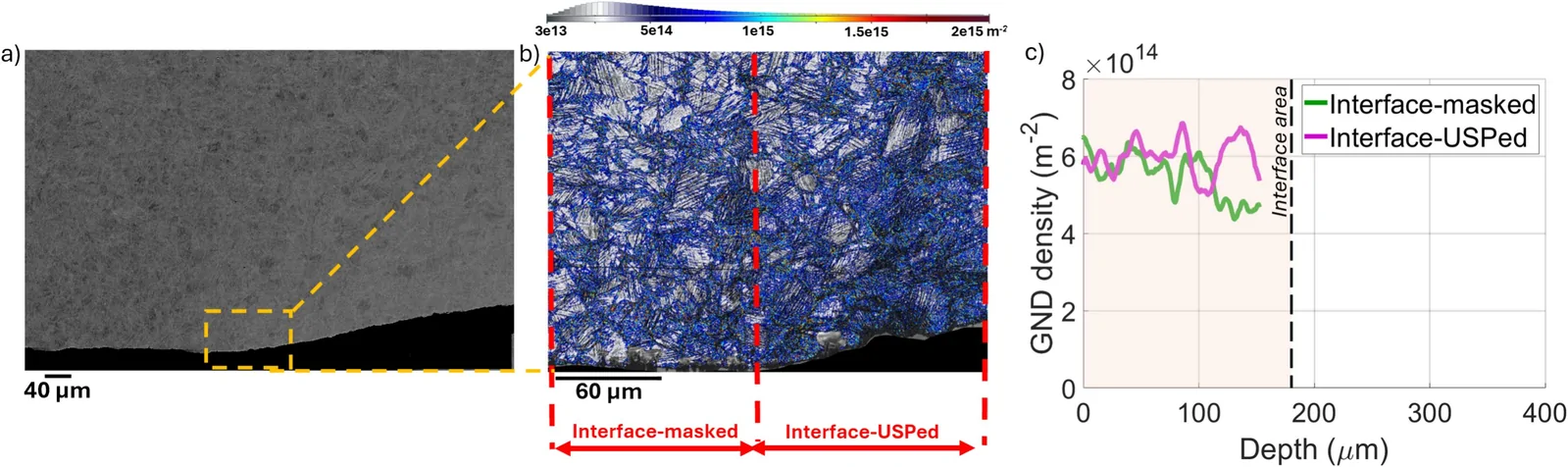
Microstructural characterization at the M-A1 locally-USPed specimen cross-section after tensile testing: a) SEM plot, b) GND map by EBSD analysis, and c) GND density gradient through depth.

To further investigate the deformation mechanisms at the interface between the USPed/masked areas after the tensile test, an EBSD analysis was performed on the same interface region of the locally-USPed M-A1 specimen analyzed before tensile testing (Fig. 6). This approach enabled a direct comparison of the GND distribution before and after tensile test. The investigated interface is shown in the SEM image in Fig. 11a, where pronounced deformation bands indicate the severe plastic deformation accumulated during tensile loading. The corresponding GND map (Fig. 11b) and the through-depth GND profiles (Fig. 11c) reveal a substantial increase in GND density across both the masked and USPed regions compared with pre-tested condition. Following tensile deformation, the GND density within the masked region increased to values comparable to the maximum GND density previously measured in the USPed region (∼7 × 10^14^ m^−2^), while the peak GND density in the USPed region was maintained at the same level and over a greater depth. This observation suggests that the pre-existing peak GND density in the USPed region may represent an apparent saturation level that didn’t change during tensile deformation; while the masked region accumulated additional GNDs until a comparable density was reached. The GNDs remained predominantly concentrated along grain boundaries, consistent with their role in accommodating plastic deformation during tensile loading. Furthermore, the masked side exhibited a GND distribution comparable to that of the USPed side, particularly within the first ∼80 µm from the treated surface. Since the pre-tested characterization (Fig. 6b) showed only limited lateral deformation beneath the mask, the additional GND accumulation after tensile testing is primarily attributed to plastic deformation under loading, while possible back-stress-driven dislocation accumulation at the soft-hard interface may also have contributed to the observed GND redistribution.

## 4 Conclusions

Mask-assisted ultrasonic shot peening was used to generate bi-directional microstructural heterogeneity in thin 316L stainless steel specimens, combining planar patterning across the surface with in-depth deformation gradients. The main findings are summarized as follows:

• Localized USP successfully produced site-specific treated and protected regions. Residual stress mapping confirmed effective masking, while SEM and EBSD analyses showed refined USPed layers, preserved microstructure beneath the masked regions, and a continuous transition at the USPed/unUSPed interfaces.
• USP induced severe plastic deformation, surface grain refinement, deformation twinning, and GND accumulation. The affected layer extended to approximately 300 µm, while martensitic transformation remained negligible under the selected processing conditions.
• Microhardness measurements through the thickness of the USPed specimens revealed a continuous hardening gradient across the entire cross-section. Sequential USP treatment generated asymmetric hardening between opposite surfaces.
• Fully-USPed specimens showed the highest strengthening but the largest ductility loss. Locally-USPed specimens exhibited intermediate strength–ductility responses between the untreated and fully-USPed conditions, confirming that the mechanical response can be modulated through the masking design.
• The reverse designs, where softer untreated regions were embedded within continuous USPed zones, showed slightly improved ductility (and slightly lower strength) and more stable strain distribution that postponed the failure, compared with the original designs.
• Increasing the USPed/unUSPed interface length alone did not significantly improve the mechanical response within the current design, likely because additional interface-induced GND accumulation was counterbalanced by the lower effective peened fraction and mask-shadowing effects.
• Microstructural characterization after tensile tests showed that the masked side of the interface between the regions accumulated GNDs to a level comparable to the peak GND density of the USPed side, suggesting an apparent saturation of GND density during tensile deformation and enhanced strain compatibility across the heterogeneous interface.

Overall, the selective USP method provides a practical route for tailoring mechanical response through controlled bi-directional microstructural heterogeneity, without changing the alloy chemical composition. As regards future developments, considering the continuous but asymmetric hardening achieved through the cross-section, the effect of consecutive steps of the treatment on the opposite faces of the thin specimens could be regarded as a design factor for future work.

## CRediT authorship contribution statement

**Erencan Oranli:** Writing – original draft, Visualization, Validation, Methodology, Investigation, Formal analysis, Data curation. **Asghar Heydari Astaraee:** Writing – review & editing, Validation, Methodology, Formal analysis. **Marc Novelli:** Writing – review & editing, Validation, Methodology, Investigation, Formal analysis. **Thierry Grosdidier:** Writing – review & editing, Supervision, Resources, Project administration, Funding acquisition, Formal analysis. **Mario Guagliano:** Writing – review & editing, Supervision, Resources, Methodology, Funding acquisition. **Sara Bagherifard:** Writing – review & editing, Supervision, Resources, Project administration, Methodology, Conceptualization.

## Declaration of competing interest

The authors declare that they have no known competing financial interests or personal relationships that could have appeared to influence the work reported in this paper.

## Data Availability

The data is already included in the mansucript
